# Identifying periodontitis risk factors through a retrospective analysis of 80 cases

**DOI:** 10.12669/pjms.38.1.5205

**Published:** 2022

**Authors:** Jing Guan, Dong Zhang, Cailing Wang

**Affiliations:** 1 Jing Guan M.D. Department of Stomatology, Hebei General Hospital; No. 348, Heping West Road, Shijiazhuang, 050051, Hebei Province, P.R. China; 2 Dong Zhang M.D. Department of Stomatology, Hebei General Hospital; No. 348, Heping West Road, Shijiazhuang, 050051, Hebei Province, P.R. China; 3 Cailing Wang M.D. Department of Stomatology, Hebei General Hospital; No. 348, Heping West Road, Shijiazhuang, 050051, Hebei Province, P.R. China

**Keywords:** Periodontitis, Risk factors, Ultrasonic scaling, Retrospective analysis

## Abstract

**Objectives:**

To investigate periodontitis risk factors and to establish a reference framework for identifying factors that place individuals at greater risk for periodontitis.

**Methods:**

Clinical data from 80 periodontitis patients admitted in the Department of Stomatology at Hebei Provincial People’s Hospital and treated between March 2020 and March 2021 were retrospectively analyzed.

**Results:**

Univariate analysis showed that lower daily brushing frequencies, decreased tooth brushing duration, decreased scaling frequency, dietary habits, smoking and drinking, genetic factors, diabetes, hypertension, and obesity were more prevalent among periodontitis patients than healthy controls. Multivariate binary logistic analysis showed that daily brushing frequency, routine scaling, smoking, drinking, heredity, diabetes, hypertension, and obesity were all risk factors for periodontitis.

**Conclusions:**

There are many risk factors for periodontitis. Clinicians need to be aware of these factors for early detection and treatment of the disease.

## INTRODUCTION

Oral health is an essential part of overall health, as well as an important indicator for the presence of other diseases. Periodontitis is one of the most common oral diseases, with an incidence second only to gingivitis. It is a chronic infection of gingival and periodontal tissue caused by bacterial plaques and biofilms.^1^ Periodontitis can destroy the integrity of tooth supporting tissue, resulting in inflammation, the formation of periodontal pockets, and alveolar bone resorption. This endangers tooth health, and is the primary cause of missing teeth in adults.^2^ In particular, individuals over 45 years of age suffer from gum atrophy. However, recent reports show that periodontitis incidence is increasing among younger individuals due to genetic, environmental, and diet-related factors.^3^ Studies have shown that the reduction or elimination of growth hormone secretion seriously inhibits the body’s absorption of vitamin C, leading to oral and gingival malnutrition.

Periodontitis has been historically difficult to cure, being very prone to relapse. Therefore, early identification and prevention of periodontitis, as well as the complete elimination of periodontitis with the aim of preventing relapse, is important. To do this, risk factors need to be identified. Previous studies indicate that periodontitis etiology is multifactorial, including heredity, living habits, and eating habits. Here, we study 80 cases of periodontitis admitted and treated at our hospital retrospectively in order to identify risk factors and establish a reference point for the prevention and treatment of periodontitis.

## METHODS

General data was obtained from 80 patients treated in the Department of Stomatology at Hebei Provincial People’s Hospital between January 2020 and March 2021. Among these 80 patients, 52 were male and 28 were female. Patients ranged between 19 to 59 years of age, with the average being 39.5±8.0 years. We also obtained data from 70 healthy controls who underwent physical examinations during the same period. This group comprised 45 males and 25 females ranging in age from 18 to 59 (average age: 37.5±7.4 years). This study was approved by the medical ethics committee of Hebei General Hospital (Approval number: 202143, approval date: 2021 July 29)

Through consultation of the established literature, a questionnaire was compiled. Information surveyed included age, gender, body mass index, smoking and drinking, oral hygiene habits, history of tooth cleaning, diabetes, blood pressure, and medical history. Data were analyzed using SPSS (v. 22.0) software. Comparisons were evaluated using chi-squared tests.

## RESULTS

Data from periodontitis patients and healthy controls were first analyzed and compared using univariate analysis. Periodontitis patients presented significantly shorter and less frequent teeth brushing sessions. Moreover, fewer periodontitis patients reported undergoing ultrasonic scaling every six months compared to the control group. Smoking and drinking was more frequent in periodontitis patients, while hereditary factors, diabetes, hypertension, and obesity were also significantly more prevalent among periodontitis patients (P<0.05, Table-I).

**Table-I:** Univariate analysis results of two groups of data.

Group	Daily brushing frequency		Brushing duration(min)		Ultrasonic scaling every six months		Smoking and drinking		Genetic factors		Diabetes		Hypertension		Obesity	

≤1	≥2	≤1	≥1.5	No	Yes	No	Yes	No	Yes	No	Yes	No	Yes	No	Yes
Study group	73	7	66	14	76	4	49	31	65	15	63	17	56	24	55	25
Control group	27	43	30	40	51	19	56	14	67	3	66	4	65	5	64	6
X²	49.395		40.259		14.1		6.25		7.397		7.484		12.507		11.711	
P	0.000		0.000		0.000		0.012		0.007		0.006		0.000		0.001	

Subsequent multivariate binary logistic analysis showed that brushing frequency (per day), presence of ultrasonic scaling performed every six months, smoking and drinking, hereditary factors, diabetes, hypertension, and obesity were all risk factors for periodontitis (P<0.05, Table-II).

**Table-II:** Multivariate binary logistic analysis of periodontitis patients.

Risk factors	B	S.E.	Wald	df	P	OR	OR 95%CI	

							Lower	Upper
Gender	1.321	0.842	2.465	1	0.116	3.748	0.72	19.505
Age	0.143	0.085	2.812	1	0.094	1.154	0.976	1.364
Brushing frequency (per day)	3.59	1.111	10.433	1	0.001	36.218	4.102	319.801
Brushing duration (min)	0.509	0.623	0.669	1	0.413	1.664	0.491	5.639
Ultrasonic scaling performed every 6 months	3.16	1.621	3.802	1	0.051	23.579	0.984	565.109
Smoking and drinking	-2.237	0.953	5.508	1	0.019	0.107	0.016	0.691
Genetic factors	-3.169	1.208	6.886	1	0.009	0.042	0.004	0.448
Diabetes	-4.606	1.314	12.292	1	0.000	0.01	0.001	0.131
Hypertension	-5.225	1.998	6.836	1	0.009	0.005	0	0.27
Obesity	-3.145	0.95	10.969	1	0.001	0.043	0.007	0.277

## DISCUSSION

Periodontitis is a chronic infectious disease of the periodontal tissue. The occurrence and development of periodontitis is not only affected by pathogenic microorganisms, but also affected by general health, individual behaviors, and societal and inherent factors. Periodontitis is a multifactorial disease closely related to oral hygiene, hereditary factors, lifestyle, diet, diabetes, blood pressure and body weight.^4^ Poor personal oral hygiene will result in oral plaque accumulation and increase the pathogenicity of subgingival plaque. This, in turn, reduces host defenses, leading to periodontitis development.^5^ This study found significant differences in the brushing habits of periodontitis patients and healthy individuals in terms of brushing frequency per day. In addition to daily oral health maintenance through brushing, flossing and other associated practices, ultrasonic scaling (commonly known as “tooth washing”) helps treat periodontitis. Here, professional medical devices remove dental calculus and subgingival plaque. As a preventive measure, individuals should undergo scaling roughly every six months.^6^ Proper dental care habits are paramount to maintaining periodontal tissue health and preventing disease occurrence or relapse.

Genetic factors are also risk factors for periodontitis. Agrawal AA et al.^7^ have found that periodontitis inflammation is largely caused by the pro-inflammatory cytokine interleukin-1 (IL-1). Differences in IL-1-related gene expression can therefore cause individuals to respond differently to the same levels of plaque. As such, certain IL-1 genotypes may serve as a risk factor for severe chronic periodontitis. Further, Nibali L, et al.^8^ conducted a meta-analysis investigating genetic factors for periodontitis, finding that the heritability (H_2_) of periodontitis was 0.38, indicating that roughly one-third of periodontitis variants may result from genetic factors. Moreover, heritability appears to increase with disease severity.^9^ The results also show that genetic factors are one of the risk factors for periodontitis.

Lifestyle habits can also endanger periodontal tissue health. Kirschneck C.^10^ showed that nicotine exposure increased periodontal bone loss and increased tooth movement velocity in an animal model. Similarly, alcohol consumption has been linked to elevated circulating neutrophil levels, which promotes periodontitis initiation through the release of reactive oxygen species intermediates, cationic peptides, and matrix metalloproteinases (MMPs).^11^,^12^ The consumption of certain foods has also been linked to periodontitis. The study found that the prevalence of smoking and drinking in periodontitis patients was higher than in the control group. In addition, Chang YC^13^ noted that betel nut chewing was linked to a higher prevalence of periodontal disease. The main chemical component of the betel nut is arecoline, which inhibits cell proliferation and reduces protein synthesis. Furthermore, nicotine has a synergistic effect on arecoline-induced cytotoxicity, meaning that certain combinations of behaviors-in this case, betel nut consumption and smoking-results in elevated periodontitis risk.

Epidemiological data presents diabetes as a major risk factor for periodontitis, with diabetics at three-fold greater risk for disease development. Along these lines, the severity of hyperglycemia has also been linked to periodontitis severity. The precise mechanism underlying this relationship is not well understood, but involves immune system function, neutrophil activity, and cytokine production.^14^ Reciprocally, Genco RJ, et al^15^ reported that periodontal disease negatively affected blood glucose control and diabetes complications. Finally, diabetes is a risk factor for tooth loss, which in turn may contribute to periodontitis etiology.^16^ This study found that more periodontitis patients had diabetes mellitus.

Hypertension is an independent risk factors of periodontitis, with potential ramifications on cardiovascular disease (CVD) complications. Hypertension, in particular, is associated with many mechanisms such as low-grade systemic inflammation and redox imbalance that render individuals more susceptible to periodontitis.^17^ In hypertensive individuals, microcirculation changes can cause periodontal tissue ischemia, a promoting factor for periodontal diseases.^18^ Conversely, studies have also shown that periodontitis can lead to increased blood pressure and decrease the effectiveness of antihypertensive therapeutics.^19^ This study also shows that hypertension is one of the independent risk factors for periodontitis, which is consistent with the literature.

Finally, increased body mass index, waist circumference, subcutaneous fat proportion, and blood lipid levels are all associated with increased periodontitis risk. The mechanisms underlying this relationship may involve adipose tissue-derived cytokines affecting systemic metabolism and creating low-grade systemic inflammation.^20^ Chaffee et al.^21^ conducted a systematic review on the relationship between chronic periodontitis and obesity, finding 41 of 70 investigated studies showing obesity to be associated with periodontitis. This study also found that the amount of tooth attachment loss was greater in obese patients with periodontitis. This study found that 25 people in the periodontitis group were obese, a much higher prevalence than the normal group.

## CONCLUSION

The findings of this study largely concur with prior reports highlighting the diversity of risk factors for periodontitis, and can serve as a reference for clinical treatment. It must be noted that this study contains a small sample size and investigates a culturally and ethnically homogenous population. Further research is necessary in the future to better clarify the relationship between various risk factors and periodontitis in order to reduce disease incidence and improve quality of life.

### Authors’ contributions:

**JG:** Conceived, designed the study and is responsible for integrity of the study.

**DZ & CW:** Collected the data and performed the analysis.

**JG:** Was involved in the Writing of the manuscript and is responsible for integrity of the study.

**JG:** Edited the manuscript.

All authors have read and approved the final manuscript.
